# Efficacy of topical cobalt chelate CTC-96 against adenovirus in a cell culture model and against adenovirus keratoconjunctivitis in a rabbit model

**DOI:** 10.1186/1471-2415-6-22

**Published:** 2006-06-05

**Authors:** Seth P Epstein, Yevgenia Y Pashinsky, David Gershon, Irene Winicov, Charlie Srivilasa, Katarina J Kristic, Penny A Asbell

**Affiliations:** 1 Department of Ophthalmology, Mount Sinai School of Medicine, New York, New York, USA; 2 Redox Pharmaceutical Corporation, Greenvale, New York, USA

## Abstract

**Background:**

Adenovirus (Ad), associated with significant morbidity, has no topical treatment. A leading CTC compound (CTC-96), a Co^III ^chelate, was found to have potent *in vitro *and *in vivo *antiviral efficacy against herpes viruses. In this study CTC-96 is being tested for possible anti-Adenovirus activity.

**Methods:**

The biological anti-adenovirus activity of CTC-96 in concentrations from 5 to 250 ug/ml, was evaluated initially by viral inactivation (viral exposure to CTC-96 followed by dilution and inoculation of cells), virucidal (viral exposure to CTC-96 and inoculation of cells without dilution) and antiviral (effect of CTC-96 on previously adsorbed virus) plaque assays on HeLa (human cervical carcinoma), A549 (human lung carcinoma) and SIRC (rabbit corneal) cells. After verifying the antiviral activity, New Zealand White rabbits were infected with Ad-5 into: 1) the anterior cul-de-sac scarifying the conjunctiva (Group "C+"); 2) the anterior cul-de-sac scarifying the conjunctiva and cornea (Group "CC+"); 3) the stroma (Group "CI+"). Controls were sham-infected ("C-", "CC-", "CI-"). Other rabbits, after "CC", were treated for 21 days with: 1) placebo, 9x/day ("-"); 2) CTC-96, 50 ug/ml, 9x/day ("50/9"); CTC-96, 50 ug/ml, 6x/day ("50/6"); CTC-96, 25 ug/ml, 6x/day ("25/6"). All animals were monitored via examination and plaque assays.

**Results:**

*In *vitro viral inactivation, virucidal and antiviral assays all demonstrated CTC-96 to be effective against Adenvirus type 5 (ad-5). The *in vivo *model of Ad keratoconjunctivitis most similar to human disease and producing highest viral yield was "CC". All eyes (6/6) developed acute conjunctivitis. "CI" yielded more stromal involvement (1/6) and iritis (5/6), but lower clinical scores (area × severity). Infection via "C" was inconsistent (4/6). Fifty (50) ug/ml was effective against Ad-5 at 6x, 9x dosings while 25 ug/ml (6x) was only marginally effective.

**Conclusion:**

CTC-96 demonstrated virucidal activity against Ad5 in tissue culture with HeLa, A549 and SIRC cell lines.

Animal Model Development: 1) "CC" produced conjunctival infection with occasional keratitis similar to human disease; "CI" yielded primarily stromal involvement; 2) "C" consistently produced neither conjunctivitis nor keratitis.

CTC Testing: 1) Conjunctivitis in all eyes; 2) Resolution fastest in "50/9" ("50/9". "50/6" > "25/6" > "-"); 3) Efficacy in "50/6" was not statistically different than "50/9"; 4) Conjunctival severity was lower in treatment groups then controls; 5) Little corneal or intra-ocular changes were noted.

## Background

Adenovirus is the most common external ocular viral infection worldwide[1]. Although not permanently blinding, ocular adenoviral infections are associated with significant patient morbidity, including symptomatic distress, and corneal changes causing visual disturbances that can last months to years. About one half of the over 50 serotypes of human adenovirus are known to cause ocular disease in patients[1]. Currently there are no specific efficacious antiviral agents for topical or systemic treatment of Adenoviral infections[2].

The studies of the pathogenesis and treatment of ocular adenovirus infections have been limited due to the narrow host range exhibited by human adenoviruses. It has been previously determined that one serotype of human adenovirus, adenovirus type 5 (Ad-5), has the ability to extend its host range to permit replication in the eyes of New Zealand rabbits[3,4]. These and other studies have shown human adenovirus type 5 (Ad-5) to present clinically within 24 to 48 hours of innoculum in rabbits[5] and last for approximately 16 days post-innoculum (mean duration of shedding)[5,6].

A number of cobalt complexes (CTC compounds) have been identified that exhibit potent *in vitro *and *in vivo *activity against herpes group viruses[7]. Most significantly, the mode of action of these novel compounds differ from currently available antiviral nucleoside analogs and protease inhibitors[8] (and unpublished data: Redox Pharmaceutical Corporation). One CTC compound in particular, CTC-96, has already been shown to exhibit pronounced efficacy in the topical therapy of HSV-1-induced epithelial and stromal disease in the rabbit eye[7].

In this study we evaluate the efficacy of topical CTC-96 against adenovirus infection in tissue culture on both human and rabbit cell lines as well as against ocular adenovirus infection in New Zealand White rabbits.

## Methods

### Materials

#### Adenovirus

Human Adenovirus type 5 [9-11] (AD-5) was obtained from the American Type Culture Collection (VR-5; ATCC, Manassas, VA) and propagated on human cervical carcinoma cell monolayers ["HeLa cells" (CCL-2); ATCC, Manassas, VA]. Stabilized viral stocks grown in EMEM [Vitacells Eagle Minimum Essential Medium (30–2003; ATCC, Manassas, VA)] supplemented with 10% fetal calf serum (FCS), 100 units/ml penicillin, 0.1 mg/ml streptomycin and 2 mM L-glutamine (all from Sigma chemical Co., St. Louis, MO) and containing approximately 9 × 10^8 ^plaque forming units/ml (pfu/ml) were produced and stored at -80°C for up to 1 year. Cells were infected at a Multiplicity of Infection (MOI) of 10. Eyes were infected with approximately 2 × 10^7 ^pfu of virus.

#### Cells

1) Human cervical carcinoma cells [HeLa cells (CCL-2); ATCC, Manassas, VA] were grown and maintained in EMEM supplemented with FCS, penicillin, streptomycin and L-glutamine as described above.

2) Human lung carcinoma cells [A549 cells (CCL-185); ATCC, Manassas, VA] were grown and maintained in Hams F-12 K Medium (30–2004; ATCC, Manassas, VA) supplemented with 10% fetal calf serum, 100 units/ml penicillin, 0.1 mg/ml streptomycin and 2 mM L-glutamine (all from Sigma chemical Co., St. Louis, MO).

3) Rabbit corneal cells [SIRC (CCL-60); ATCC, Manassas, VA] were grown and maintained in EMEM supplemented with 10% fetal bovine serum, 100 units/ml penicillin, 0.1 mg/ml streptomycin and 2 mM L-glutamine (all from Sigma chemical Co., St. Louis, MO).

#### Pharmaceuticals

**Figure 1:**
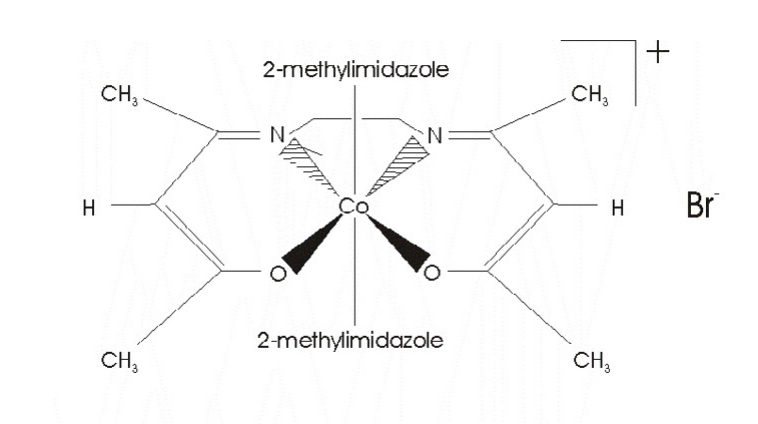
The general structure of Doxovir™ (CTC-96).

Doxovir™, CTC-96 (Figure 1) was synthesized according to a standard Redox Pharmaceutical Corporation procedure[12]. CTC-96 solution (stable in aqueous solutions at 4°C for >6 months and for several weeks at 37°C) was prepared from crystalline compound which is completely stable for over 2.5 years when stored desiccated at 0°C. The structure was confirmed by the manufacturer utilizing ^1^H and ^13^C nuclear magnetic resonance spectroscopy, mass spectrometry, and elemental analysis[12]. Using these techniques, purity was determined to be ≥ 99%, with no detectable impurities[12]. Prior to use, drug concentrations in all CTC-96 solutions were confirmed by HPLC against an independently prepared CTC-96 standard. The activity of the drug solutions was confirmed in an anti-Herpes Simplex-1 assay[9]. Formulations of this stabilized active compound at the appropriate concentrations were prepared, filter-sterilized and supplied by the manufacturer. Samples were stored refrigerated and protected from light prior to use.

#### Animals

New Zealand White Rabbits weighing 4–5 pounds were purchased from H.A.R.E. Industries (Hewitt, NJ). Animals were designated both SPF (specific pathogen free) and VAF (viral antigen free) and were handled in accordance with NIH guidelines. At the conclusion of each experiment, the rabbits were euthanized by pentobarbital injection (Sleepaway, Fort Dodge Labs, Fort Dodge, IA).

Rabbits were housed in the pathogen containment area (BSL-2) of the Mount Sinai Medical Center Animal Facility. Newly received animals were allowed to acclimate to the facility and to daily handling for one week prior to infection. All rabbits were examined to verify no clinical or culture evidence of viral disease (slit lamp biomicroscopy and viral cultures of corneal swabs followed by standardized plaque assays).

### Procedures

#### I. *In vitro *studies

##### A. *In vitro *viral inactivation assays (direct drug-virus {high titer} neutralization with post-neutralization CTC dilution)

Varying concentrations of CTC-96 or placebo were mixed with concentrated human Adenovirus type 5 (Ad5; 1.75 × 10^7 ^pfu) and incubated at 37°C for 60 minutes (min) under atmospheric conditions. Aliquots of the virus/drug suspension were then diluted 500 fold and plated in triplicate on aspirated HeLa, A549 and SIRC cell monolayers to initiate infection (6–8 × 10^5 ^cells/cm2 in T-25 culture flasks containing 9.9 mls fresh culture medium giving a total volume of 10 mls with an MOI of 10). The low final concentration of the CTC-96 (1:500) has no inhibitory effect on Adenovirus type 5 growth. All cell lines were grown and maintained in their appropriate medium.

The cell monolayers, having been infected with the virus, were incubated for 24 hours (hr) at 37°C and 5% CO2 and then washed, scraped, sonicated, centrifuged and the supernatant serially diluted. These serial dilutions were plated onto indicator HeLa, A549 and SIRC cell monolayers and adsorbed for 60 min. The supernatant was then aspirated and a methylcellulose overlay placed over the cells which were then incubated at 37°C. Due to differences in replication rates of the Adenovirus in the various cell lines, HeLa cells were incubated for 3 days, A549 cells for 5 days and the SIRC cells for 7 days. All were counterstained with 1% methylene blue, allowed to dry and the plaques counted under a phase contrast microscope in a masked fashion.

##### B. *In vitro *virucidal assays (direct drug-virus neutralization without post-neutralization CTC dilution)

Varying concentrations of CTC-96 or placebo were mixed with concentrated human Adenovirus type 5 (Ad5; 1.75 × 10^7 ^pfu) and incubated at 37°C for 60 minutes (min). Media of the HeLa, A549 and SIRC cell monolayers (at 80% confluency) was decanted and replaced with the above suspension. These cell monolayers, having now been infected with the virus (6–8 × 10^5 ^cells/cm2 in T-25 culture flasks containing 9.9 mls fresh culture medium giving a total volume of 10 mls with an MOI of 10), were incubated for 24 hours (hr) at 37°C and 5% CO_2 _in medium containing CTC-96 at the indicated concentrations and then washed, scraped, sonicated, centrifuged and the supernatant serially diluted.

The serial dilutions were then plated onto indicator HeLa, A549 and SIRC cell monolayers and adsorbed for 60 min as described above. The supernatant was then aspirated and a methylcellulose overlay placed over the cells which were then incubated at 37°C. All were counterstained with 1% methylene blue, allowed to dry and the plaques counted under a phase contrast microscope in a masked fashion.

##### C. *In vitro *antiviral activity assays (effect of CTC-96 on virus previously adsorbed onto cells)

Adenovirus type 5 (1.75 × 10^7 ^pfu) was adsorbed onto preconfluent HeLa, A549 and SIRC cell monolayers for 60 min at 37°C. Various concentrations of CTC-96 or placebo in medium were then added to the culture and the monolayers were subsequently incubated for 24 hr at 37°C and 5% CO_2_. Monolayers were then washed, scraped, sonicated, centrifuged, the supernatants serially diluted and ultimately stained and the plaques counted as described above under the section for the viral inactivation assay.

##### D. Procedure

The biological anti-adenovirus activity of freshly solubilized CTC-96 was evaluated by the standard antiviral, virucidal and antiviral plaque reduction assays as described above. Seven concentrations of CTC-96 were used with each cell line and in each assay: 0, 5, 10, 25, 50, 100 and 250 ug/ml. Both negative (0 ug/ml CTC-96, no Ad-5) and positive [0 ug/ml CTC-96, addition of Ad-5 (MOI = 10)] controls were included. All were diluted in the appropriate medium for each of the cell lines.

##### E. Data analysis

All clinical and virus recovery data were analyzed for statistical differences, utilizing the mean ± standard deviation for each of the various groups in computer-generated two-tailed bivariant Student's t tests (GB-STAT, New England Software, Inc., College Station, TX, U.S.A.; SAS, SAS Institute Inc., Cary, NC, U.S.A.; and SPSS, SPSS Inc., Chicago, IL, U.S.A.)[13,14]. Individual Fisher exact tests were also performed (GB-STAT, SAS and SPSS)[13,14], as well as an overall chi-squared analysis (GB-STAT, SAS and SPSS)[13,14] and a correlation coefficient[13,14]. Two-tailed significance was established at a confidence level of 0.05 ≤ P ≤ 0.95.

#### II. Animal studies

##### A. Methods of viral infection (6 groups)

1. Experimental

a. Installation of 20 ul containing 4 × 10^7 ^pfu Ad-5 into the anterior cul-de-sac and scarifying the conjunctiva (4 scratches) with a 25-gauge needle (Group "C+": 8 eyes).

b. Installation of 20 ul containing 4 × 10^7 ^pfu Ad-5 into the anterior cul-de-sac and scarifying the conjunctiva and cornea (8 scratches: 4 to conjunctiva, 4 to cornea) with a 25-gauge needle (Group "CC+": 8 eyes).

c. Injection of 20 ul containing 4 × 10^7 ^pfu Ad-5 into the corneal stroma with a 25-gauge needle (Group "CI+": 8 eyes).

Sham-infected animals of all groups were attempted as controls:

2. Controls

a. Installation of 20 ul containing sterile HeLa media [Eagles Minimum Essential Medium (EMEM; ATCC, Manassas, VA) + 10% fetal calf serum (Hyclone, Logan, UT) + 1% penicillin/streptomycin (Sigma Chemical Company, St. Louis, MO) + 1% l-glutamine (Sigma Chemical Company, St. Louis, MO)] into the anterior cul-de-sac and scarifying the conjunctiva (4 scratches) with a 25-gauge needle (Group "C-": 8 eyes).

b. Installation of 20 ul containing sterile HeLa media into the anterior cul-de-sac and scarifying the conjunctiva and cornea (8 scratches: 4 to conjunctiva, 4 to cornea) with a 25-gauge needle (Group "CC-": 8 eyes).

c. Injection of 20 ul containing HeLa media into the corneal stroma with a 25-gauge needle (Group "CI-": 8 eyes).

3. Procedure

The eyes were randomly sorted into groups, examined by a masked observer (ophthalmologist) by slit-lamp biomicroscopy [Days 1 (preinfection), 3, 5, 7, 10, 12, 15, 17, 19, 23, 26, 30 post-infection (pi)] and scored using the grading system for rabbit conjunctival disease (Table 1) derived from that originally described by Wander *et al*[15] for herpes simplex virus (HSV).

**Table 1:** *In Vitro*Plaque Assays. Human Adenovirus type 5 (Ad-5) viral titers [plaque forming units (pfu)/preliminary flask] in Virucidal, Antiviral and Viral Inactivation assays in HeLa (human cervical carcinoma), A549 (human cervical carcinoma) and SIRC (rabbit corneal) cells.

**Com-pound**	**Cell Line**	**Conc.**	**Virucidal**			**Antiviral**			**Viral Inactivation**		
			**Average**		**Standard Deviation**	**Average**		**Standard Deviation**	**Average**		**Standard Deviation**

		**(μg/ml)**	**(PFU)**		**(PFU)**	**(PFU)**		**(PFU)**	**(PFU)**		**(PFU)**

Negative Control (None)	HeLa	0	0.00 × 10^0^	±	0.00 × 10^0^	0.00 × 10^0^	±	0.00 × 10^0^	0.00 × 10^0^	±	0.00 × 10^0^
	A549	0	0.00 × 10^0^	±	0.00 × 10^0^	0.00 × 10^0^	±	0.00 × 10^0^	0.00 × 10^0^	±	0.00 × 10^0^
	SIRC	0	0.00 × 10^0^	±	0.00 × 10^0^	0.00 × 10^0^	±	0.00 × 10^0^	0.00 × 10^0^	±	0.00 × 10^0^
CTC-96	HeLa (human cervix)	0	5.00 × 10^9^	±	0.00 × 10^0^	4.00 × 10^10^	±	0.00 × 10^0^	5.33 × 10^9^	±	5.77 × 10^8^
		5	4.67 × 10^9^	±	5.77 × 10^8^	1.77 × 10^10^	±	5.77 × 10^8^	4.67 × 10^9^	±	5.77 × 10^8^
		10	3.67 × 10^9^	±	5.77 × 10^8^	1.33 × 10^10^	±	5.77 × 10^8^	2.47 × 10^9^	±	5.77 × 10^7^
		25	3.67 × 10^9^	±	5.77 × 10^8^	1.47 × 10^9^	±	5.77 × 10^7^	1.17 × 10^9^	±	5.77 × 10^7^
		50	6.67 × 10^1^	±	5.77 × 10^1^	1.00 × 10^2^	±	1.00 × 10^2^	3.33 × 10^1^	±	5.77 × 10^1^
		100	0.00 × 10^0^	±	0.00 × 10^0^	0.00 × 10^0^	±	0.00 × 10^0^	0.00 × 10^0^	±	0.00 × 10^0^
		250	0.00 × 10^0^	±	0.00 × 10^0^	0.00 × 10^0^	±	0.00 × 10^0^	0.00 × 10^0^	±	0.00 × 10^0^
	A549 (human lung)	0	3.07 × 10^9^	±	5.77 × 10^7^	2.47 × 10^10^	±	5.77 × 10^8^	3.33E+09	±	5.77 × 10^8^
		5	2.77 × 10^9^	±	5.77 × 10^7^	1.17 × 10^10^	±	5.77 × 10^8^	2.77E+09	±	5.77 × 10^7^
		10	2.27 × 10^9^	±	5.77 × 10^7^	8.67 × 10^9^	±	5.77 × 10^8^	1.47E+09	±	5.77 × 10^7^
		25	2.10 × 10^9^	±	0.00 × 10^0^	9.33 × 10^7^	±	5.77 × 10^6^	6.67E+08	±	5.77 × 10^7^
		50	6.67 × 10^1^	±	5.77 × 10^1^	6.67 × 10^1^	±	5.77 × 10^1^	3.33E+01	±	5.77 × 10^1^
		100	0.00 × 10^0^	±	0.00 × 10^0^	0.00 × 10^0^	±	0.00 × 10^0^	0.00 × 10^0^	±	0.00 × 10^0^
		250	0.00 × 10^0^	±	0.00 × 10^0^	0.00 × 10^0^	±	0.00 × 10^0^	0.00 × 10^0^	±	0.00 × 10^0^
	SIRC (rabbit cornea)	0	5.67 × 10^8^	±	5.77 × 10^7^	3.67 × 10^9^	±	5.77 × 10^8^	5.33 × 10^8^	±	5.77 × 10^7^
		5	4.67 × 10^8^	±	5.77 × 10^7^	3.67 × 10^9^	±	5.77 × 10^8^	3.00 × 10^8^	±	5.77 × 10^7^
		10	4.33 × 10^8^	±	5.77 × 10^7^	3.10 × 10^9^	±	1.00 × 10^8^	3.33 × 10^8^	±	5.77 × 10^7^
		25	5.77 × 10^7^	±	0.00 × 10^0^	2.03 × 10^9^	±	5.77 × 10^7^	1.00 × 10^7^	±	0.00 × 10^0^
		50	3.33 × 10^1^	±	5.77 × 10^1^	3.33 × 10^1^	±	5.77 × 10^1^	0.00 × 10^0^	±	0.00 × 10^0^
		100	0.00 × 10^0^	±	0.00 × 10^0^	0.00 × 10^0^	±	0.00 × 10^0^	0.00 × 10^0^	±	0.00 × 10^0^
		250	0.00 × 10^0^	±	0.00 × 10^0^	0.00 × 10^0^	±	0.00 × 10^0^	0.00 × 10^0^	±	0.00 × 10^0^

Corneal swabs (Viral Culturettes, Baxter, Deerfield, IL) were taken for viral plaque assay twice per week [Days 1 (preinfection), 3, 5, 7, 10, 12, 15, 17, 19, 23, 26 and 30 pi]. Briefly, sterile culturettes were gently swabbed across the corneas (one per cornea) and the swabbed culturettes immediately eluted with 0.5 ml of Hanks balanced salt solution and serial 1:10 dilutions prepared for virus assays. Neither sterile culturettes nor cultures received any other manipulation. Culturettes were not frozen prior to titration. Plaque assays were performed in triplicate to ensure reproducibility.

##### B. CTC testing

1. Infection

The conjunctivae and corneas were infected with 20 ul containing 4 × 10^7 ^pfu of virus into the anterior cul-de-sac and then scarified with a 25-gauge needle (8 scratches: 4 to conjunctiva, 4 to cornea: exactly as in Group "CC+" above)[16].

2. Treatment Groups (4 groups)

The animals were sorted into treatment groups of matched disease severities on day 7 post-infection, when all eyes showed disease signs, averaging 2–3, suggestive of acute conjunctivitis.

Grouped animals were treated, both eyes identically, with instillation of a 20 ul drop per eye of:

a. Placebo treatment (formulation without active ingredient), nine times per day [9x/day ("-"): 2 eyes],

b. CTC-96, 50 ug/ml, 9x/day ("50/9"), 2 eyes,

c. CTC-96, 50 ug/ml, six times per day [6x/day ("50/6")], 2 eyes,

or

d. CTC-96, 25 ug/ml, 6x/day ("25/6"), 2 eyes.

The formulation consisted of: 45 mg/ml mannitol, 60 ug/ml benzalkonium chloride, 2 mg/ml 2-methylimidazole in aqueous solution (pH 7.4). Drug treatments were spread out over each day (7:30 a.m. to 9:30 p.m.), for 21 days of treatment (both eyes of each animal treated identically).

3. Procedure

The eyes were examined in a masked fashion by an ophthalmologist. They were graded clinically (Table 1) and corneal swabs (Viral Culturettes, Baxter, Deerfield, IL) were taken for viral plaque assay twice per week [Days 7 (pretreatment on day of treatment initiation), 10, 14, 17, 20, 25, 28, 31, 35 and 38 post-infection (pi; Days 1, 3, 7, 10, 13, 18, 21, 24, 28 and 31 post-treatment initiation (pti)]. Briefly, sterile culturettes were gently swabbed across the corneas (one per cornea) and the swabbed culturettes immediately eluted with 0.5 ml of Hanks balanced salt solution and serial 1:10 dilutions prepared for virus assays. Neither sterile culturettes nor cultures received any other manipulation. Culturettes were not frozen prior to titration. Plaque assays were performed in triplicate to ensure reproducibility.

a. Standardized Viral Plaque Assays

Titers of active virions of adenovirus, scored on preconfluent HeLa cell culture monolayers and expressed as plaque forming units (pfus) were determined according to established procedures[17]. Monolayers were grown and maintained in "HeLa media" [Vitacell= s Eagle Minimum Essential Medium (30–2003; ATCC, Manassas, VA) + 10% fetal calf serum + 1% penicillin/streptomycin + 1% l-glutamine (all from Sigma chemical Co., St. Louis, MO). Three days after inoculation (at 37°C and 5% CO_2_) with serial ten-fold dilutions of analyte, plaques had formed and the plates were stained with 1% methylene blue, allowed to dry and the plaques counted under a phase contrast microscope in a masked fashion in that the examiner had no knowledge of the treatment protocol for the individual animals. The animals were examined in a unique, random order for each examination.

b. Ocular Grading System

Clinical disease was divided up into: conjunctival, corneal epithelial and stromal disease and iritis. Corneal epithelial disease was subdivided into SPK, epithelial adenovirus symptoms, pannus, and epithelial defect. Stromal disease was subdivided into: edema, melting, neovascularization and infiltrate. Both the area and severity of each subdivision was graded individually from 0 to +4. Area of involvement was represented in increasing amounts of 25% (0 = "normal cornea", +1 = ≤ 25%, +2 = >25%, ≤ 50%, +3 = > 50%, ≤ 75%, +4 = > 75%, ≤ 100%). While severity of each subdivision was graded while the severity of each subdivision was individually graded from 0 (normal cornea) to +4 (severe).

c. Data Analysis

aa. Clinical Data

While conjunctival, corneal epithelial, stromal and iritis data was collected clinically, for simplicity only conjunctival data was graphed as other findings were insignificant (≈0). Clinical scores (area × severity) were calculated and utilized for all statistical calculations.

Non parametric methods of statistical analysis were used because disease severity is graded by assigning numerical scores[15,16] to observed lesions. The arithmetic means of such arbitrarily scaled scores should not be analyzed by parametic methods such as the student's t test. Instead, rank medians[18,19], a Wilcoxson-Mann-Whitney rank-sum test[19,20], as well as both Spearman[19,21] and Kendall[19,22] rank correlations, as per standard non-parametric statistics, were used for analysis of the rank nonparametric assigned scores because non-parametric statistical techniques permit evaluation of significance of chemotherapeutic efficacies when measured by changes in severity of disease signs. The results presented were considered significant at a rank median of ≤ 0.05 units.

bb. Virus Recovery Data

Viral titers (per ml) were analyzed for statistical differences, utilizing the mean ± standard deviation for each of the various groups in computer-generated two-tailed bivariant Student's t tests[13,14] (GB-STAT, New England Software, Inc., College Station, TX, U.S.A.; SAS, SAS Institute Inc., Cary, NC, U.S.A.; and SPSS, SPSS Inc., Chicago, IL, U.S.A.)[13,14] at all time points. Individual Fisher exact tests[13,14] were also performed, as well as an overall chi-squared analysis[13,14] and a correlation coefficient[13,14]. Two-tailed significance was established at a confidence level of 0.05 ≥ P ≥ 0.95.

## Results

### I. *In vitro *studies

Viral inactivation, virucidal and antiviral efficacy studies in tissue culture demonstrated CTC-96 to be effective against human Adenovirus type 5 in a dose-dependent fashion (Tables 1 and 2). CTC-96 at doses ≥ 50 ug/ml shows high levels of antiviral efficacy in the viral inactivation, virucidal and antiviral (post-infection) models, against human Adenovirus type 5 in all three cell lines used. The standardized Viral Inactivation, Virucidal and Antiviral assays showed viral yield to be almost zero at a concentration of 50 ug/ml and virtually zero at 100 ug/ml for the two human cell types [HeLa & A549; virucidal and antiviral: 6.67 × 10^1 ^pfu/flask (8-fold inactivation of virions: Table 1)]. In rabbit corneal cells (SIRC), viral yield reached undetectable levels even at 50 ug/ml (Tables 1 and 2). There was a precipitous, dose dependent inhibition between 25 and 50 ug/ml of CTC-96 and a modest effect at still lower concentrations (i.e. 2.47 × 10^9 ^pfu at 10 ug/ml and 1.17 × 10^9 ^pfu at 25 ug/ml; Table 1) demonstrating, as previously mentioned, dose dependence.

**Table 2:** ED 50s & 90s of Doxovir™ to Adenovirus Type 5. Effective dose (ED) 50s and 90s of Doxovir™ (CTC-96) against Adenovirus type 5 in HeLa (human cervical carcinoma), A549 (human cervical carcinoma) and SIRC (rabbit corneal) cells during Virucidal, Antiviral and Viral Inactivation assays.

		(MIC 50 (μg/ml)			(MIC 90 (μg/ml)		
**HeLa (human cervical carcinoma)**	**Virucidal**	33.0	±	6.9	46.6	±	9.7
	**Antiviral**	4.5	±	0.1	21.8	±	1.2
	**Viral Inactivation**	16.5	±	2.5	43.0	±	1.3
**A549 (human lung carcinoma)**	**Virucidal**	31.7	±	1.9	46.3	±	2.6
	**Antiviral**	4.7	±	0.2	20.8	±	1.9
	**Viral Inactivation**	4.2	±	1.1	37.5	±	6.5
**SIRC (rabbit cornea)**	**Virucidal**	13.6	±	2.3	22.7	±	3.5
	**Antiviral**	27.3	±	8.3	45.5	±	1.6
	**Viral Inactivation**	13.1	±	2.9	23.9	±	4.9

In all assays, the compound exhibited consistently effective antiviral effects against adenoviral infections in HeLa, A549 and SIRC cell types (Tables 1 and 2) and statistically significant differences were observed in all assays (Viral Inactivation, Virucidal and Antiviral) between each of the three cell types. These differences were fairly constant for the various cell lines. For example, A549 viral titers were consistently approximately half (i.e. approx 50%) those of the identical assays run on HeLa cells (see Table 1), while the titers of the rabbit corneal cells (SIRC) were consistently one sixth (i.e. approx 17%) those of the identical assays run on human lung carcinoma (A549; see Table 1).

### II. Animal studies

#### A. Methods of viral infection

**Figure 2:**
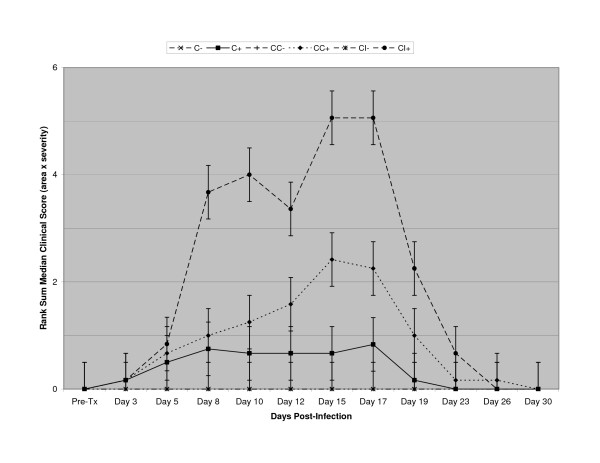
Pearson rank sum medians ± nonparametric equivalents of standard deviation of conjunctival clinical scores (clinical score = area × severity) of conjunctivae of model development animals by group. Group "C": corneal infection, conjunctival scarification; Group "CC": corneal infection, conjunctival and corneal scarification; Group "CI": stromal infection. "+" animals ("C+", "CC+", "CI+") were infected with active virus, "-" were sham-infected ("C-", "CC-", "CI-").

Clinical biomicroscopic examination and standardized antiviral plaque assay showed rabbit ocular infection with human Adenvirus type 5 via the corneal and conjunctival scarification method (Group "CC") produced an acute conjunctivitis (6/6 eyes) with occasional keratitis that appeared similar to human disease (Figure 2). The CI group produced lower conjunctival clinical scores than the CC group, but did yield rare stromal involvement (1/6 eyes) and greater iritis (5/6 eyes; Figure 2). Infection via the conjunctival scarification method (Group "C+") yielded conjunctivitis in 4/6 eyes (Figure 2). None of the control animals ("C-", "CC-", "CI-") developed any signs of conjunctivitis or keratitis.

**Figure 3:**
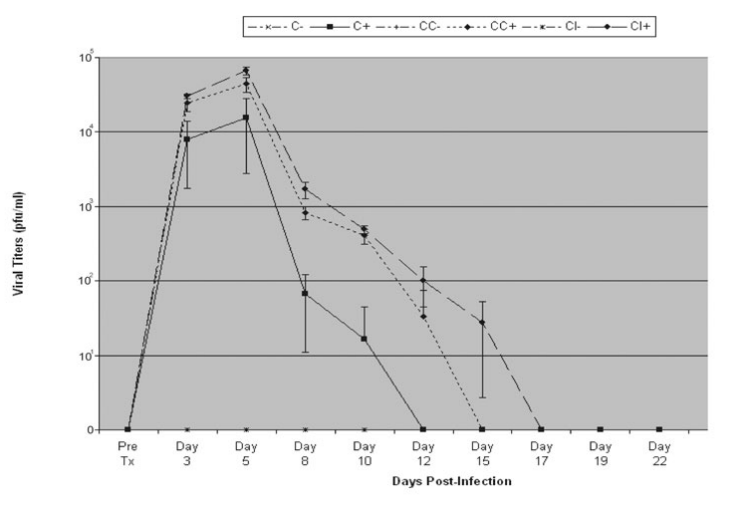
Averages ± standard deviations of infectious virus titers [plaque forming units (pfu)/ml] of model development animals by group. Group "C": corneal infection, conjunctival scarification; Group "CC": corneal infection, conjunctival and corneal scarification; Group "CI": stromal infection. "+" animals ("C+", "CC+", "CI+") were infected with active virus, "-" were sham-infected ("C-", "CC-", "CI-").

The plaque assays matched the clinical disease (Figure 3). The corneal and conjunctival scarification group (CC) developed viral titers by day 3 post-infection comparable to those of the corneal injection group (CI; Figure 3). Bearing this out, the conjunctival scarification group (C) developed viral titers significantly less (other than on day 5 p.i.) than either of the other two groups (Figure 3). While viral clearance was achieved in the conjunctival scarification group (C) at day 12 p.i., it was not achieved until day 15 p.i. for CC or 17 for CI (Figure 3) in the other two.

Since the corneal and conjunctival scarification group (CC) consistently developed acute conjunctivitis similar to human disease this model was chosen to evaluate the CTC treatment for acute adenovirus conjunctivitis.

#### B. CTC testing

Both clinical biomicroscopic examination and standardized antiviral plaque assay showed CTC-96 at all concentrations and frequencies tested was effective against ocular disease caused by adenovirus type 5. The majority of clinical signs of infection were in the conjunctiva, with only minimal and occasional signs observable in the corneal epithelium and/or stroma.

**Figure 4:**
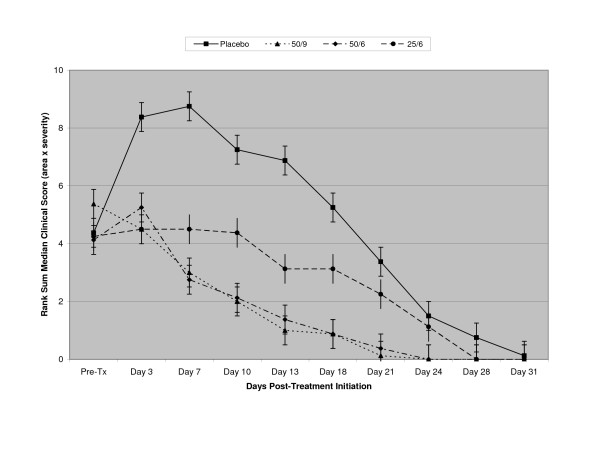
Pearson rank sum medians ± nonparametric equivalents of standard deviations of conjunctival clinical scores (clinical score = area × severity) of CTC-treated animals by group. Placebo (squares, solid line); 50 ug/ml, 9x/day ["50/9" (triangles, dotted line)]; 50 ug/ml, 6x/day ["50/6" (diamonds, mixed solid/dotted line)]; 258 ug/ml, 6x/day ["25/6" (asterisks/circles, dashed line)].

Clinically, placebo-treated eyes initially showed a gradual worsening in clinical scores (up to a rank sum of 8) until Day 14 pi (Day 7 pti), after which they slowly resolved for another 3 weeks (Figure 4).

Animals treated with CTC-96 at 50 ug/ml, showed a steady improvement in clinical scores (Figure 4) in both of the dosing frequencies evaluated (9x/day and 6x/day). No adverse events were noted in any of the eyes and the clinical scores did not exceed 4–5 with these treatments. Statistical significance as compared to the placebo group was achieved for [Days 10, 14, 17, 20, 25 and 28 pi (Days 1, 3, 7, 10, 13, 18 and 21 pti; 50/9: Wilcoxin-Mann: p ≤ 0.03; 50/6: Wilcoxin-Mann: p ≤ 0.04). The marginal effect that the increased frequency of 9x/day appeared to have had over that of 6x/day was not statistically significant. Clinically, conjunctivae of both frequencies (9x/day and 6x/day: Figure 4) of CTC-96 at 50 ug/ml resolved on Days 28 – 31 pi (Days 21–24 pti) and were statistically indistinguishable in clinical severity until resolution (Wilcoxin-Mann: p ≥ 0.80).

In all cases, CTC-96 at 25 ug/ml, 6x/day, was effective against human adenovirus type 5 as compared to sham-treated eyes. Although only statistically significant at Days 10, 14 and 20 pi (Days 3, 7 and 13 pti), while the placebo-treated eyes were still visibly resolving clinically at Day 38 pi (the last exam day: Day31 pti), CTC-96 at 25 ug/ml, 6x/day, had visibly resolved by Day 35 (Day 28 pti; Figure 4).

**Figure 5:**
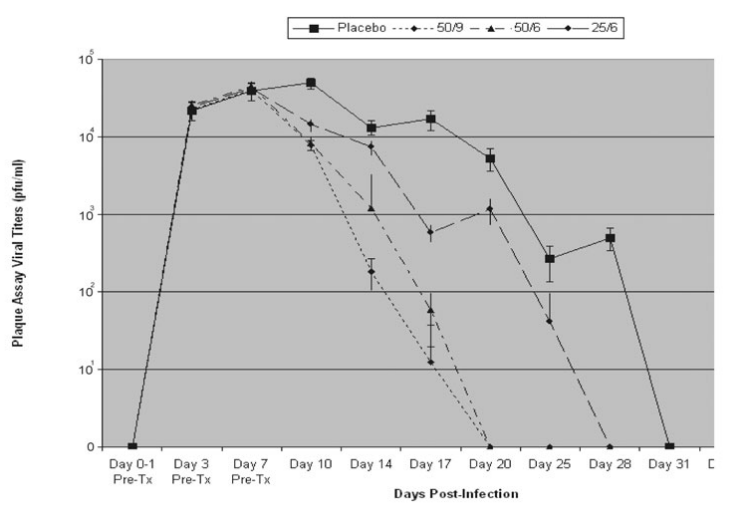
Averages ± standard deviations of plaque assay viral titers [plaque forming units (pfu)/ml] of CTC-treated animals by group. Placebo (squares, solid line); 50 ug/ml, 9x/day ["50/9" (triangles, dotted line)]; 50 ug/ml, 6x/day ["50/6" (diamonds, mixed solid/dotted line)]; 258 ug/ml, 6x/day ["25/6" (asterisks/circles, dashed line)].

The clinical observations in which placebo-treated eyes initially showed a gradual worsening, after which they slowly resolved, was confirmed by the plaque assays (Figure 5)]. Viral clearance was not achieved until Day 28 pi (Day 21 pti; Figure 5) in the placebo-treated eyes.

The viral plaque assays also demonstrated that CTC-96 at 50 ug/ml in both of the dosing frequencies evaluated (9x/day and 6x/day), eliminated virus by Day 20 pi (Day 13 pti; Figure 5), whereas 25 ug/ml, 6x/day was significantly less effective [t test: p ≥ 0.01 (50 ug/ml, 9x/d > 50 ug/ml, 6x/d: p ≥ 0.01; 25 ug/ml: p = 0.02)]. While viral clearance occurred at Day 20 pi (Day 13 pti) at both of the dosage frequencies of 50 ug/ml (Figure 5), viral titers were already significantly lowered (ie there was a significant difference between the two) at Day 10 pi (Day 3 pti) as compared to that of the control [t test: p = 0.02 (50 ug/ml, 9x/d < 50 ug/ml, 6x/d)].

At 25 ug/ml, 6x/day, CTC-96 was effective against human adenovirus type 5 as compared to placebo-treated eyes and viral clearance in this group was not achieved until Day 28 pi (Day 21 pti), as compared to Day 31 pi (Day 24 pti) in the placebo-treated eyes (Figure 5). Thus, in conclusion, although some therapeutic effects of CTC-96 at 25 ug/ml are observed at 6x/day, it was considerably inferior to 50 ug/ml (50 ug/ml, 9x/day. 50 ug/ml, 6x/day >> 25 ug/ml, 6x/day).

## Discussion

This study demonstrates that the cobalt chelate CTC-96 (designated Doxovir™) is an effective antiviral agent against human Adenovirus type 5 both in cell culture and in rabbits. Adenovirus type 5 infection of human and rabbits cells was effectively treated by CTC-96 and primary ocular human adenovirus type 5-induced conjunctivitis in a New Zealand White rabbit model was effectively treated by topical administration with CTC-96. Treatment considerably reduced both disease severity and corneal surface viral titers, significantly shortening the course of the disease and the symptoms in parallel. The data presented here indicate that against Ad-5, six (6) daily applications of CTC-96 at 50 ug/ml are approximately as effective as nine (9) daily applications. Six (6) daily applications of 25 ug/ml CTC-96 are inferior to either of the other two, but significantly better than placebo. It is most likely that this demonstrates a dose responsiveness, although the possibility of a low sample population size producing the observed effect cannot be entirely discounted.

Currently there are no specific antiviral agents for topical or systemic treatment of adenoviral infections[2]. Antiviral agents active against HSV and interferons have been used in the treatment of Adenovirus infections with only limited effect[23,24]. Thus far, previous recent *in vitro*experiments with the cobalt chelate CTC-96, in addition to showing strong anti-herpes simplex efficacy[7,8], have demonstrated anti-human adenovirus type 5 efficacy of CTC-96[5,6]. In addition, newer nucleoside analogs have recently been found to exhibit some inhibitory activity against Adenovirus *in vitro*and *in vivo *[24-27].

Topical cidofovir, a nucleoside analog, was shown to be an effective topical agent against against adenovirus in non-human systems and may have some effect in man[28,29], however it has proven to be too toxic in human eyes. Interestingly, despite the relatively short experience with the drug, cidofovir-resistant adenovirus variants have already been observed[30]. Since CTC-96 has been found to have a very different mechanism of action than nucleoside analogs, it has been shown to be effective against drug-resistant mutants of herpes viruses (unpublished results: Redox Pharmaceutical Corporation).

In the past it has been difficult to define a reliable animal model of adenovirus ocular disease. For the most part previous studies of the pathogenesis and treatment of ocular adenovirus infections have been limited by the narrow host range exhibited by human adenoviruses. About half of the greater than 50 serotypes of human adenovirus are known to cause ocular disease in humans [3-6] yet only three have been previously found to have the ability to extend their host range to permit replication in the eyes of New Zealand rabbits [3-6].

We developed a simple, reliable method of inducing adenovirus infection that mimics human disease using a rabbit model. Previous animal models of adenovirus employed the injection of active virus into the corneal stroma first described by Gordon, *et al*[3]. This "corneal injection method" is technically difficult and neither mimics nor even approximates the usual routes of human infection. For this reason, we evaluated three (3) methods of infecting the ocular surface with adenovirus to determine which most mimicked human adenovirus ocular disease, and was repeatable and easy to create: 1) the "conjunctival scarification method" involving the installation of 20 ul containing 2 × 10^7 ^pfu Ad-5 into the anterior cul-de-sac with a scarified conjunctiva (Group "C+"); 2) the "corneal and conjunctival scarification method" involving the installation of 20 ul containing 2 × 10^7 ^pfu Ad-5 into the anterior cul-de-sac after both the conjunctiva and cornea were scarified (Group "CC+"); and 3) the "corneal injection method" involving the intrastromal injection of 20 ul containing 2 × 10^7 ^pfu of Ad-5 into the cornea (Group "CI+"). Concurrent controls with conjunctival scarification (Group "C-") or corneal and conjunctival scarification (Group "CC-") did not develop acute conjunctivitis or keratitis as evaluated by a masked observer.

When compared both clinically and via the quantification of viral titers (standardized plaque assay), infection via the corneal and conjunctival scarification method (Group "CC") produced a conjunctival infection with occasional keratitis most similar to human disease. It was easier and more reliable than the corneal injection (Group "CI") or the conjunctival scarification methods. The corneal injection method utilized by other authors[3,4] yields more stromal involvement than the "CC" group ["CI": 2/6 eyes (33%) versus "CC": 0/6 eyes (0%)] but is more difficult to create.

The results presented here demonstrate that adenovirus infection via the corneal and conjunctival scarification method (Group "CC") produced the best rabbit model to study conjunctival infection, while the corneal injection method (Group "CI") produced more stromal involvement and iritis making it the model of choice to study stromal disease and iritis.

As previously mentioned, a number of cobalt complexes (CTC compounds) were found to exhibit potent *in vitro *and *in vivo *activity against herpes group viruses [7]. The CTC compound in this study, CTC-96, has already been shown to exhibit pronounced efficacy in the treatment of HSV-1 in cell culture studies on vero cells[7] as well as in topical therapy of HSV-1-induced epithelial and stromal disease in the rabbit eye[7]. Safety data and toxicity studies have shown the agent to exhibit minimal signs of toxicity and to not be readily absorbed during topical application[7,8] (and unpublished data: Redox Pharmaceutical Corporation). This previous research discounted the possibility that the effect of CTC-96 is due to toxicity by evaluating the effects of CTC-96 both in tissue culture [5–500 ug/ml (no toxicity ≤50 ug/ml, trace and transient at 100 ug/ml): Vero (African Green monkey kidney fibroblast cell line), HeLa (human cervical cancer cell line), A549 (human alveolar carcinoma cell line), SIRC (immortalized rabbit corneal epithelial cell line) and HeP2 (human laryngeal squamous cancer cell line)[5-8] and unpublished observations] and systemic applications (IP and IV) in mice (unpublished observations), as well as via topical administration of 20 ul drops 3x, 4x, 5x, 6x and 9x/day on normal uninfected rabbit (New Zealand White rabbits) eyes [total of 240 eyes,10–500 ug/ml, checked clinically and histologically[7] (and unpublished observations)] although it is still possible that diseased eyes may be more sensitive than normal ones. Phase II studies for the treatment of HSV-1 are presently underway.

There is a noticeable difference in the efficacious dose against Adenovirus than that against Herpesvirus, where concentrations as low as 5 ug/ml were found to possess strong antiviral activity *in vitro*[7] and in rabbit eyes with an IC_50 _for HSV-1 *in vitro*of 0.7 ug/ml (Winicov, Redox unpublished observations). Current findings on human Adenovirus type 5 (Ad-5) demonstrated that a concentration of 50 ug/ml of CTC-96 is required to obtain full antiviral efficacy. We attribute at least part of this difference to the effect of the drug on membrane proteins of enveloped viruses (e.g. HSV-1 and HIV) rather than capsid or intra-capsid proteins. CTC-96 interacts with a membrane glycoprotein of HIV that results in virus inactivation (in preparation). Since Adenovirus does not possess a membrane, it is possible that the inhibitory action of the drug involves binding to one or more capsid or intra-capsid proteins that are less accessible to the drug than membrane surface proteins in enveloped viruses.

Thus, CTC-96 has a completely different mode of action than nucleotide analogs as well as other DNA replication inhibitors. The virucidal effect of CTC-96 can be due to various modes of action of the drug, but the major activity of the drug entails disruption of protein conformation through binding to methyl imidazole nitrogens of specific histidines in several protein types[31]. But the compound is also known to be a superoxide scavenger[32] (and unpublished data: Redox Pharmaceutical Corporation).

In conclusion, the corneal and conjunctival scarification method of adenovirus infection has proven to be a usable model of ocular Ad-5 infection in the rabbit as it approximates the signs and symptoms of adenvirus-derived conjunctivitis in human eyes. In addition, topical CTC-96 has demonstrated significant anti-viral activity against human adenovirus type 5 (Ad-5) in these *in vivo *models both by clinical examination and viral plaque assays.

Clearly more studies will be needed to evaluate completely the efficacy and safety of CTC-96 and clinical trials in humans will be necessary to determine the safety and efficacy of CTC-96 for treating human adenoviral keratoconjunctivitis.

## Conclusion

### I. *In vitro *studies

A. No toxic effects observed in HeLa, A549 or SIRC cells for CTC-96 at the therapeutic concentration of 10 ug/ml.

B. CTC-96 demonstrated virucidal activity against Adenovirus type 5 in tissue culture with HeLa, A549 and SIRC cell lines.

1. In all cases, peak efficacy was observed in concentrations ≥ 50 μg/ml, while lower concentrations showed decreasing dose-responsiveness.

2. Greater viral replication/yield and antiviral activity was observed in HeLa >A549 > SIRC.

### II. Animal studies

#### A. In the Model Development experimentation (testing of the method of infection)

1. Infection via corneal and conjunctival scarification method (Group "CC") produced a conjunctival infection with occasional keratitis similar to human disease.

2. The corneal injection method (Group "CI") yielded primarily stromal involvement.

3. The conjunctival scarification method (Group "C") did not consistently produce acute conjunctivitis.

4. The conjunctival scarification method (Group "C") did not produce any keratitis.

#### B. In the CTC Efficacy experimentation (testing of the efficacy of human adenovirus type 5 in a NZW rabbit model)

1. Conjunctivitis was seen in all eyes by day 8 post-infection.

2. Resolution was fastest in treatment group "50/9" where most animals resolved by Day 21 post-treatment initiation (pti):

"50/9" ≈ "50/6" > "25/6" > "-".

2. The efficacy of CTC-96 in treatment group A50/6" was not statistically less effective than that observed in treatment group A50/9": most animals resolving by Day 21 pti.

3. The degree of severity of the conjunctiva was lower in all treatment groups as compared to the control eyes.

4. Little corneal or intra-ocular changes were noted in any of the treatment groups.

## Competing interests

Supported by Redox Pharmaceutical Corp., grants from Research to Prevent Blindness, Inc., New York, NY; a research grant from Turobiner-Finley Grant Fund, New York, NY; and in part by EY01867 from the National Eye Institute, National Institutes of Health, Bethesda, MD.

## Authors' contributions

SPE participated in the design, experimentation and drafted the manuscript. YYP participated in the experimentation. DG conceived of the study with PAA. IW participated in the coordination of the study. CS participated in the experimentation. KJK participated in the experimentation. PAA conceived of the study with DG. All authors read and approved the final manuscript.

## Pre-publication history

The pre-publication history for this paper can be accessed here:


